# Injection of a Bone Substitute in the Treatment of Unicameral Bone Cysts

**DOI:** 10.1155/2023/3270372

**Published:** 2023-01-05

**Authors:** B. Sivakumar, V. V. G. An, A. Dobbe, D. Drynan, D. Little

**Affiliations:** ^1^Department of Orthopaedics, Westmead Childrens' Hospital, Hawkesbury Rd, Westmead, Sydney, Australia; ^2^Royal North Shore Hospital, Pacific Hwy, St Leonards, Sydney, Australia

## Abstract

**Background:**

Simple bone cysts are benign bony lesions. Treatment strategies are varied for this particular pathology. It remains controversial as to what the ideal treatment strategy is. Recently, bony substitute injections have emerged as a potential option for treatment. This paper aimed to describe our institution's experience in using bony substitute injections to treat unicameral bone cysts.

**Methods:**

A retrospective review of consecutive patients over an 84-month period at a tertiary paediatric hospital was performed. Information regarding patients' presentation, diagnosis, and management was recorded and summarised.

**Results:**

A total of 15 patients were included in our study, with a mean follow-up of 118 weeks. 86.7% of patients demonstrated clinical resolution (absence of pain at the latest follow-up) and 80% of patients demonstrated radiographic resolution. Only one patient sustained a subtrochanteric fracture post-index operation, whilst two others demonstrated redevelopment of cystic architecture on follow-up.

**Conclusion:**

This study demonstrates that bone substitute injection is potentially a minimally invasive and seemingly successful technique in the treatment of unicameral bone cysts and other simple bone lesions. Further randomised and comparative studies are required to confirm and validate our findings.

## 1. Introduction

Simple bone cysts (also known as solitary or unicameral bone cysts) are benign lesions that were first described by Virchow in 1876 [1]. They are fluid-filled, single-chamber lesions situated in the metaphyses of long bones in children and adolescents and constitute approximately 3% of all bone lesions. Although often resolving after skeletal maturity, they predispose children to fractures, can be painful, and may restrict function due to concerns regarding refracture or upon following medical advice–thus, treatment is often used to speed resolution [2]. Fractures through bone cysts in the femoral neck may result in avascular necrosis, and some bone cysts, or their treatment, can result in significant growth disturbance [3].

Treatment strategies are varied and may involve a combination of intralesional injections, decompression and bone grafting, damage to the cyst's lining, and structural stabilization. Substances that may be injected include steroids (such as methylprednisolone acetate), autologous bone marrow, demineralized bone matrix, and bone substitutes (calcium sulphate, calcium phosphate, or a combination of both) [4–8]. There is little agreement as to the ideal substance for intralesional injection due to clinical equipoise regarding the long-term results of each treatment strategy. In particular, there are limited data available on the outcomes of bone substitute injections for the treatment of unicameral cysts [9–13].

Thus, the aim of this study is to report the authors' results for the treatment of unicameral bone cysts with bone substitute injections (BSI), review the available literature, and provide recommendations for the future.

## 2. Methods

This study was performed as a retrospective review of a series of consecutive patients at a tertiary-referral paediatric hospital in Sydney, Australia. After obtaining institutional ethics approval, records of all patients treated for unicameral bone cysts with the injection of a bone substitute material (either with curettage alone or accompanied by skeletal fixation) were obtained for an 84-month period (August 2009–August 2016). Patient details were identified by searching the computerized medical record system utilizing key search terms.

Medical charts and radiographs were then reviewed to adjudge suitability for inclusion. Patients with unicameral bone cysts, bone substitute injections, preoperative films, and clinical and radiographic follow-ups were included. Those with alternate lesions or injections, or inadequate clinical or radiographic documentation were excluded.

Demographic information and baseline lesion characteristics were collected, including location, cyst activity, and cyst index. A cyst is considered active if it is adjacent to the cartilaginous growth plate and inactive if it migrated away and is separated by normal cancellous bone [14]. The cyst index gives an indication of the size of the cyst relative to the involved bone and is calculated as ((area of the cyst)/(diaphyseal diameter2)) [15]. The diaphyseal diameter is measured at its tubular section, whilst the area of cyst is calculated by its largest radiological dimensions (either on the lateral or anteroposterior radiograph).

The primary outcome assessed was the complete resolution of the simple bone cyst at follow-up, and it was assessed both clinically and radiographically. Secondary outcomes included complications (such as growth disturbance or fracture) and the presence of pain. Outcomes were independently appraised by two investigators (BSS and VVGA) and the consensus was met on any disagreements regarding the interpretation of radiographs or clinic notes.

## 3. Results

Over the 84-month period, 28 patient records were identified during the medical record search as having had a simple bone cyst treated with an injection of a bone substitute. Of these, 8 patients were excluded for having alternate pathology, 4 were excluded due to inadequate pre-operative or postoperative films, and a duplicate record was removed, leaving 15 patients eligible for inclusion.

The majority of the patients were male, with a mean age of 6.7 years old [2, 4–9, 16, 17] at presentation (refer Table 1). One patient was skeletally mature at the time of initial cyst detection. All cysts were located in weight-bearing bones, with one in the calcaneus and the remainder in the proximal femur. 10 patients presented due to a pathological fracture. The majority of cysts were active (11; 78.6%), with 3 inactive and one skeletally mature patient. The mean cyst index was 2.7 (1.2–4.8, SD = 0.94).

**Figure 1:**
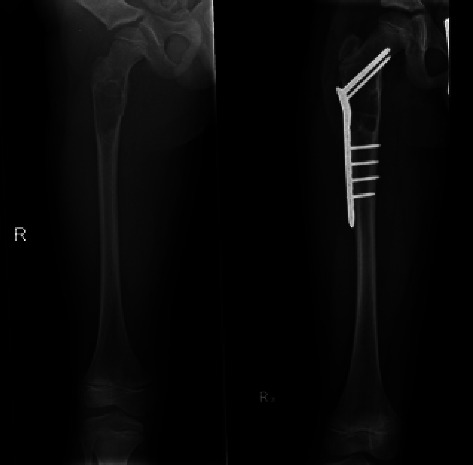
Preoperative images (medial and lateral) of a patient who received open reduction and internal fixation with bone substitute injection.

**Figure 2:**
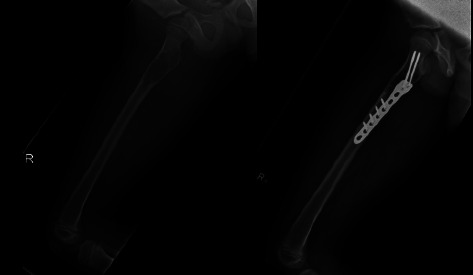
Postoperative images (medial and lateral) of a patient who received open reduction and internal fixation with bone substitute injection.

2 patients had initial internal stabilization with curettage alone, with a revision of fixation and injection of bone substitute for recurrence of a cyst 112 and 348 weeks later, respectively. Of the remainder, 5 patients underwent internal stabilization with paediatric blade plates with curettage and BSI (Figures 1 and 2), whilst the remainder had curettage and BSI alone. A single patient had an injection of Pro-Dense (Wright Medical Group, Middlesex, UK) which is a combination of calcium sulphate and calcium phosphate, whilst Hydroset (Stryker, Kalamazoo, Michigan, USA), containing calcium phosphate alone, was utilized in the rest.

Follow-up was performed with the serial clinical and radiographic examination. The mean follow-up was 118 weeks (6–288). Clinical resolution (defined as the absence of pain on palpation) was seen in 13 patients (86.7%), whilst radiographic resolution (defined as the complete absence of cystic architecture at final follow-up) was seen in 12 patients (80.0%). One patient with initial curettage and BSI (cyst index 3.2) sustained a subtrochanteric fracture at 107 weeks post-index operation. The fracture was plated, with partial radiographic resolution at the latest follow-up. 2 other patients demonstrated redevelopment of cystic architecture on follow-up radiographs, with one patient undergoing re-injection of bone substitute at 45 weeks post index operation, resulting in complete clinical and radiographic resolution. A patient further underwent re-operation for revision fixation due to prominent hardware (refer to Table 2).

## 4. Discussion

Treatment strategies for unicameral bone cysts are varied, with all forms of treatment historically associated with high levels of recurrence [9]. Substances utilized in intralesional injections include steroids (such as methylprednisolone acetate), autologous bone marrow, demineralized bone matrix, and bone substitutes (calcium sulphate, calcium phosphate, or a combination of both) [4–8]. There is little agreement as to the ideal substance for intralesional injection due to clinical equipoise regarding the long-term results of each treatment strategy.

Methylprednisolone acetate injections were the “traditional” form of treatment. Although Scaglietti initially described healing rates of up to 90%, more recent studies had reported lower resolution of between 33% and 41%, with the need for multiple injections [4, 9, 17–19]. Similarly, early results of bone marrow injections were extremely promising, demonstrating a 100% rate of healing after one injection; however, subsequent studies have shown lower rates [5, 20–23]. A randomised trial showed methylprednisolone to be superior to bone marrow aspirate, but recurrence was over 50% in each group, illustrating that neither treatment produced truly favourable results [2].

Although autologous bone remains the optimal graft, concerns regarding limited supply, donor site morbidity, and invasive implantation have resulted in the increasing popularity of bone substitutes [24–26]. Furthermore, the results of using autologous grafting in simple bone cysts are still poor, possibly due to excessive resorption [2, 5, 9, 20, 27]. Calcium sulphate (CaSO_4_) and calcium phosphate (CaPO_4_) are osteoconductive, biodegradable, and percutaneously injectable, particularly in the paediatric population [28]. They exhibit different resorption, mechanical, and side effect profiles; calcium sulphate is more acidic and dissolves quicker, resulting in a higher frequency of long-term drainage and subsequent wound complications, whilst calcium phosphate maintains its architecture for a longer duration but has been associated with adverse and sometimes painful soft tissue reactions [29–34]. Composites have been developed to improve compressive strength and generate intermediate resorption [10, 28].

The major substitutes available in Australia are Hydroset, Pro-Dense, and Beta-BSM (Zimmer Biomet, Warsaw, Indiana, United States). Hydroset is a temperature-sensitive calcium phosphate cement that is prepared by mixing a liquid and powder together to achieve a homogenous, consistent injectable paste. Setting time varies between 8 and 10 minutes from the start of mixing, and the graft proceeds to precipitate the formation of hydroxyapatite [35]. Pro-Dense is a composite of calcium phosphate and calcium sulphate and is prepared similarly to result in an injectable graft that sets in approximately 30 minutes [36]. Pro-Dense has FDA approval for injection into benign bone cysts. Beta BSM uses proprietary nanocrystalline calcium phosphate technology, and the contents are undisclosed.

Bohner classified all calcium phosphate cements (CPCs) into either apatite-forming or brushite-forming [37]. He noted that brushite-forming CPC resorbed earlier than apatite-forming CPC in sheep bone defects. Signs of inflammation or immunologic response leading to delayed new bone formation were not noticed at any time. Apelt et al. confirmed that brushite CPC resorbed much faster than apatite-forming cement [38].

The mechanism behind the effect of BSI in causing the resolution of unicameral bone cysts is uncertain. Acidic breakdown products may be unfavourable to cells responsible for the prolongation of cyst architecture; however, this remains conjecture. The osteoconductive nature of these materials may also stimulate bone ingrowth and resolution of the lesion [39].

There is limited evidence regarding long-term efficacy of bone substitute utilization in simple bone cysts. Hou et al. reported healing rates of 66% for curettage and calcium sulphate implantation and 91% for a combination of curettage, ethanol cauterization, cyst membrane disruption, and calcium sulphate grafting [9]. Dormans et al. demonstrated healing rates of 90% with minimally invasive curettage and calcium sulphate pellet grafting [11]. Similarly, Mohamad et al. reported a healing rate of 92% using calcium phosphate bone substitute in humeral lesions with 4 year follow-up [12]. Composites of both calcium sulphate and phosphate demonstrate similarly high rates of the resolution, with rates of approximately 93% reported [10, 13].

This study demonstrates complete radiographic resolution after one injection in 80% of patients, with clinical resolution in 87%. It adds to and correlates with the available literature purporting that BSI is a minimally invasive and seemingly successful technique in the treatment of unicameral bone cysts and other simple bone lesions. It also indicates that patients with a greater cyst index. However, this study (and the others available) is not without its deficiencies. Radiographic outcomes are based on plain films, which may result in difficulty adjudging resolution due to the radio-opaque nature of bone substitute materials. The treating surgical team conducted all observations and analyses, which could contribute to measurement bias. Further, the retrospective nature of these studies, combined with small patient numbers, can weaken any interpretation of cause and effect. Currently, no level-one evidence exists to support their use; thus, further long-term results and comparative studies are required. It should also be noted that none of the cysts included in this study were in the proximal humerus–which is the most common location for UBCs. The role of curettage and bone substitutes needs to be elucidated in this population.

## 5. Conclusion

This study demonstrates that bony substitute injection is potentially a safe, minimally invasive, and safe technique in the treatment of unicameral bone cysts. However, further studies with randomised controlled trials and other comparative forms of study are required to confirm our findings and consolidate the evidence base for its use.

## Figures and Tables

**Table 1:** Baseline characteristics.

	Values (standard deviation or percent)
Age at diagnosis	6.7 (2.4)
Sex (male/female)	12/3 (80.0%/20.0%)
Cyst index	2.7 (0.94)
Cyst active	11 (78.6%)
Fracture at presentation	10 (66.7%)

**Table 2:** Postoperative characteristics.

	Values (standard deviation or percent)
Follow-up (weeks)	117.7 (SD)
Reoperation	3 (20.0%)
Complete clinical resolution	13 (86.7%)
Complete radiographic resolution	12 (80.0%)

## Data Availability

Raw data for this study were unfortunately unable to be provided, as this paper was a small case series. Providing data would be potentially identifiable to patients. However, the data and conclusions presented are basic summary statistics.
